# Supplementary material for: Hydrophobin Gene Cmhyd4 Negatively Regulates Fruiting Body Development in Edible Fungi Cordyceps militaris
Source: Int J Mol Sci. 2023 Feb 27;24(5):4586. doi: 10.3390/ijms24054586 (PMC10003708; doi:10.3390/ijms24054586)

Hydrophobin gene *Cmhyd4* negatively regulates fruiting body development in edible fungi *Cordyceps militaris*

Xiao Li[1, 2], **Mengqian Liu**[1, 3], **Caihong Dong**[1*]

[1]State Key Laboratory of Mycology, Institute of Microbiology, Chinese Academy of Sciences, Beijing, 100101, China; [2]College of Horticulture, Hebei Agricultural University, Baoding 071001, China; [3]University of Chinese Academy of Sciences, Beijing 101408, China;

lixmushroom@gmail.com (XL); liumengqian1011@gmail.com (MQL); dongch@im.ac.cn (CHD)

*Corresponding author

Caihong Dong

State Key Laboratory of Mycology

Institute of Microbiology, Chinese Academy of Sciences

NO.3 Park 1, Beichen West Road, Chaoyang District, Beijing, 100101 China

E-mail: dongch@im.ac.cn

Figure S1. The relative transcript levels of fruiting body development related genes at the primordium stage in the *ΔCmhyd4* strain. All results were based on the standard levels of the MY stage of *ΔCmhyd4* strain (ΔCmhyd4-MY). Error bars indicate the standard deviation (SD) of three biological replicates with three technical replicates.

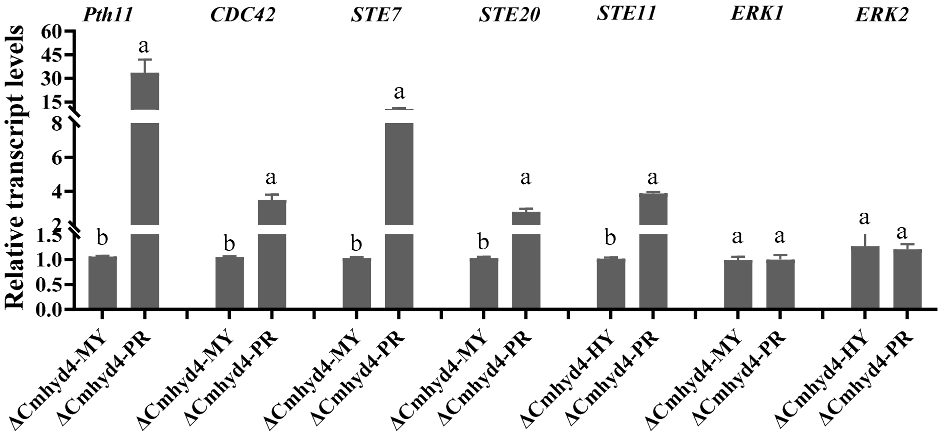

Supplement: Supplementary file 1 [file ijms-24-04586-s001.zip › Supplementary file 2.pdf]
